# Artificial intelligence-driven new drug discovery targeting serine/threonine kinase 33 for cancer treatment

**DOI:** 10.1186/s12935-023-03176-2

**Published:** 2023-12-12

**Authors:** Na Ly Tran, Hyerim Kim, Cheol-Hee Shin, Eun Ko, Seung Ja Oh

**Affiliations:** 1 Program in Nanoscience and Technology, Graduate School of Convergence Science and Technology, Seoul National University, Seoul, Republic of Korea; 2 Department of Genetics and Biotechnology, College of Life Sciences, Kyung Hee University, Yongin-Si, 17104 Gyeonggi-Do Korea; 3 Center for Biomaterials, Korea Institute of Science and Technology (KIST), Biomedical Research Institute, Seoul, Republic of Korea; 4 Standigm Inc, Seoul, Republic of Korea

**Keywords:** Artificial intelligence, Drug repurposing, Serine/threonine kinase 33, Cancer treatment, Virtual screening

## Abstract

**Background:**

Artificial intelligence (AI) is capable of integrating a large amount of related information to predict therapeutic relationships such as disease treatment with known drugs, gene expression, and drug-target binding. AI has gained increasing attention as a promising tool for next-generation drug development.

**Methods:**

An AI method was used for drug repurposing and target identification for cancer. Among 8 survived candidates after background checking, *N*-(1-propyl-1H-1,3-benzodiazol-2-yl)-3-(pyrrolidine-1-sulfonyl) benzamide (Z29077885) was newly selected as an new anti-cancer drug, and the anti-cancer efficacy of Z29077885 was confirmed using cell viability, western blot, cell cycle, apoptosis assay in MDA-MB 231 and A549 in vitro. Then, anti-tumor efficacy of Z29077885 was validated in an in vivo A549 xenograft in BALB/c nude mice.

**Results:**

First, we discovered an antiviral agent, Z29077885, as a new anticancer drug candidate using the AI deep learning method. Next, we demonstrated that Z29077885 inhibits Serine/threonine kinase 33 (STK33) enzymatic function in vitro and showed the anticancer efficacy in various cancer cells. Then, we found enhanced apoptosis via S-phase cell cycle arrest as the mechanism underlying the anticancer efficacy of Z29077885 in both lung and breast cancer cells. Finally, we confirmed the anti-tumor efficacy of Z29077885 in an in vivo A549 xenograft.

**Conclusions:**

In this study, we used an AI-driven screening strategy to find a novel anticancer medication targeting STK33 that triggers cancer cell apoptosis and cell cycle arrest at the s phase. It will pave a way to efficiently discover new anticancer drugs.

**Supplementary Information:**

The online version contains supplementary material available at 10.1186/s12935-023-03176-2.

## Introduction

Computational drug repurposing is an effective strategy for determining new applications of existing drugs [1, 2]. Drug repurposing generally starts with a computational screening of pre-existing compounds, which employs various computational methods such as ligand similarity analysis, molecular docking, and machine learning. In particular, artificial intelligence (AI) deep learning technology capable of integrating a large amount of related information to predict therapeutic relationships has been proposed as a next-generation drug development methodology [3, 4]. Traditionally, the development of new drugs, from target identification to FDA approval, requires more than 10 years and is cost-intensive [5]. This is a significant hurdle for both academia and biotechnology companies. Therefore, drug repurposing using AI methods can be a tangible approach for cost- and time-effective drug development [6]. Serine/threonine kinase 33 (STK33) is a member of the calcium/calmodulin-dependent kinase family and is implicated in various cancers [7]. STK33 is involved in cancer initiation, progression, and resistance to therapy [8, 9]. It has also been suggested as a potential therapeutic target in patients with *KRAS*-mutant cancer [10–12]. Various inhibitors of STK33 have been developed, and kinase-dependent and -independent roles of STK33 in malignant tumors have been demonstrated [13, 14]. For example, BRD-8899 was developed as a potent and selective inhibitor of STK33. Even though BRD-8899 could inhibit kinase activity, it did not show anticancer efficacy in KRAS-dependent cells [13]. Additionally, other STK33 inhibitors, including ML-281, failed to rescue the survival rate of KRAS-dependent cancer cells [14]. Although the therapeutic potential of inhibiting STK33 in patients with *KRAS*-mutant cancer is controversial, STK33 remains an attractive therapeutic target in several other cancers. However, STK33-targeting cancer therapeutics have not been approved for patient-use yet.

In this study, we identified a new anti-cancer drug targeting STK33 that induces apoptosis and cell cycle arrest at s phase by AI. First, we screened drug candidates that have never been reported as anticancer drugs, from public open chemical libraries using the AI deep learning method, and discovered Z29077885, an antiviral agent, as an anticancer drug candidate. Next, we found that Z29077885 inhibits STK33 enzymatic function in vitro. Also, we demonstrated the anticancer efficacy of Z29077885 in various cancer cells. Then, we found enhanced apoptosis and s phase cell cycle arrest as the mechanism underlying the anticancer efficacy related to Z29077885. Finally, the anti-tumor potency of Z29077885 was demonstrated in an in vivo A549 xenograft. Taken together, this study shows Z29077885, repurposed through virtual screening and with anticancer potency both in vitro and in vivo, could be a new promising therapeutics against cancer.

## Materials and methods

### Human cell lines

THP-1, A549, MCF-7, 253 J, SNU790, U87MG, SKOV3, HepG2, Hela Caco2, MKN45, MDA-MB-231 and A375SM cell lines were purchased from either the Korean Cell Line Bank (Seoul, Korea). NOMO-1 and SKM-1 were purchased from Japanese Collection of Research Bioresources Cell Bank (Osaka, Japan). All cell lines were maintained in Eagle's minimum essential medium and Roswell Park Memorial Institute medium supplemented with 10% FBS and antibiotics in a humidified atmosphere with 5% CO_2_ at 37 ℃.

### Chemicals

N-(1-propyl-1H-1,3-benzodiazol-2-yl)-3-(pyrrolidine-1-sulfonyl) benzamide (Z29077885) was purchased from Enamine Ltd (Ukraine, CAS#785710-21-6). BRD-8899 and ML-281 (#4880), STK33 inhibitors, were purchased from Medchem and Tocris, respectively.

### Viability assay

Cancer cells were treated with STK33-23 and incubated for 72 h. The cell viability was measured using CellTiter-Glo Luminescent Cell Viability Assay (Promega, Cat#G7570) by GloMax Discover Multimode Microplate Reader (Promega, Madison, WI, USA).

### Anti-cancer effect of STK33-23 in 3D breast cancer model

MDA-MB 231, BT-474, SKBR3, and MCF-7 spheroids were made by adding 2000 cells in 100uL of RPMI per well on Ultra-low attachment 96 plates (Costar, #Cat7007) and centrifuged 1000 rpm for 2 min after that 100 μL of 5% Matrigel solution in RPMI (Corning, Cat#354234) was gently added to the plate in order to obtain 2.5% Matrigel media as a final solution. The plate was centrifuged one more time at 1000 rpm for 2 min and incubated for 48 h at 37 °C in a humidified atmosphere with 5% CO_2_. After 48 h, spheroid was treated with STK33-23 with varying concentrations and confirmed viability by Cell Titer Glo assay (Promega, #CatG7572) after 72 h of treatment.

### Apoptosis measurement by AnexinV/7AAD flow cytometry

MDA-MB 231 and A549 were seeded 0.5 × 10^6^ cells per well in 6 well plates after 12 h, 50 µM and 100 µM of STK33-23 was treated and incubated for 24 h. Cells were detached by dissociation buffer (Millipore, #S01413) and centrifuged 2000 rpm for 2 min, then removed supernatant. Cells were washed one time with cold FACS buffer (500 mL PBS, 5 ml FBS, 5 mL P/s, 1 mM EDTA) and resuspend in 1X Binding Buffer (BD). 5 µl of APC Annexin V (BD, Cat#5504774) and 2 µl of 7-AAD (BD, Cat#51-68981E) were added in cells and incubated for 15 min at RT (25 °C) in the dark. 1X Binding Buffer was added to each tube, and stained cells were analyzed by flow cytometry within 1 h. The samples were included unstained cells, cells stained with APC Annexin V alone (no 7-AAD), and cells stained with 7-AAD alone (no APC Annexin V) to set up compensation. Data were analyzed by FlowJo software.

### Cell cycle assay by PI flow cytometry

MDA-MB 231 and A549 were seeded 0.5 × 10^6^ cells per well in 6 well-plate. After 12 h, 50 µM and 100 µM of STK33-23 were treated and incubated for 24 h. Cells were detached by dissociation buffer. All of the cell media and PBS wash were kept and pelleted with cells media and PBS wash at 2000 rpm for 2 min. Cells were washed by PBS and pelleted again at 2000 rpm for 2 min. The cell was fixed with 400 µL of 66% cold ethanol on ice, stored 4 degrees for at least 2 h. Cells were pelleted again at 2000 rpm for 2 min and carefully removed supernatant. Cells were washed one more time with 1X PBS. The cells were re suspended in 200 µL of 1X PI (Abcam Cat#139418) (200 µL per 1 staining is the dilution of 100 µL of 20X PI + 10 µL of 200X RNase in 1890 mL). Stained cells were incubated at 37 degrees in the dark for 30 min. Place the tube on ice and go for flow cytometry. Cell cycles were analyzed by FlowJo software.

### Protein analysis

For protein analysis, the cells were disrupted by RIPA buffer containing protease/phosphotase inhibitor cocktail (Abcam). The amount of total protein in the supernatant was determined by Pierce™ BCA Protein Assay Kit (Thermo Scientific). Samples were boiled at 95 °C for 5 min in SDS loading sample buffer (Biosesang), separated by SDS-PAGE and transferred onto a polyvinylidene difluoride (PVDF) (Bio-rad) followed by blocking with 5% BSA in TBST (TBS with 0.1% Tween 20) for phosphoprotein and 5% skim milk in TBST for total protein at room temperature 30 min, blocking was repeated twice. The membrane was incubated with primary antibodies P-STAT3 (9145S), STAT3(9139S), GAPDH(97166S) from Cell Signaling in TBST containing 5% BSA or 5% skim milk at 4 °C overnight. Secondary antibody anti-rabbit or anti-mouse HRP-conjugated (Cell Signaling) was blotted at room temperature for 1 h. The immunoblots were visualized by Amersham™ ECL™ Prime Western blotting detection reagent, and then luminescent images were obtained using iBright™ (Invitrogen).

### In vivo study

All the animal care and in vivo experiments in this study were followed by the regulations of the Institutional Animal Care and Use Committee of KIST. BALB/c nude mice (6 weeks, male) were purchased from ORIENT BIO Inc. For the generation of the A549 xenografts, 3 × 10^7^ cells / 50 µl Opti-MEM media were subcutaneously inoculated into the flanks of mice. The drug treatment was performed when tumor volumes reached approximately 100–150 mm^3^. For the anti-cancer effect of STK33-23, the drug was injected intratumorally at a dose of 2 mg/50 µl (DMSO 10%, PEG-400 90%). The control group was treated with 50 µl of DMSO 10% and PEG-400 90% solution. All treatments were performed every 4 days three times. The tumor size was measured every 4 days and determined by the formula V = (A x B^2^)/2, in which A is the longer diameter and B is the shorter length.

### Artificial Intelligence-aided drug repurposing

Drug repurposing candidates for cancer were obtained by Standigm Insight™, an AI technology for drug repurposing and target identification. After validating the efficacies of the proposed candidates, we predicted and analyzed potential mechanisms of action of the compounds which showed therapeutic effects for cancer. Standigm Insight™ contains a massive knowledge graph database established by integrating thirty public databases and manually curated information for explaining the therapeutic pattern between a compound and a disease. Additionally, Standigm Insignt™ also incorporates a target prioritization AI module to predict potential binding targets for given compounds.

### Statistical analysis

All data were expressed as mean value ± standard deviation, and each experiment was performed in triplicate for three separate experiments. Data were analyzed using a one-way analysis of variance (ANOVA) followed by Tukey’s post hoc test. The statistical significance was *p < 0.05, **p < 0.01, and ***p < 0.001.

## Results

### Identification of the antiviral drug Z29077885 as a new anticancer drug targeting STK33

**Fig. 1:**
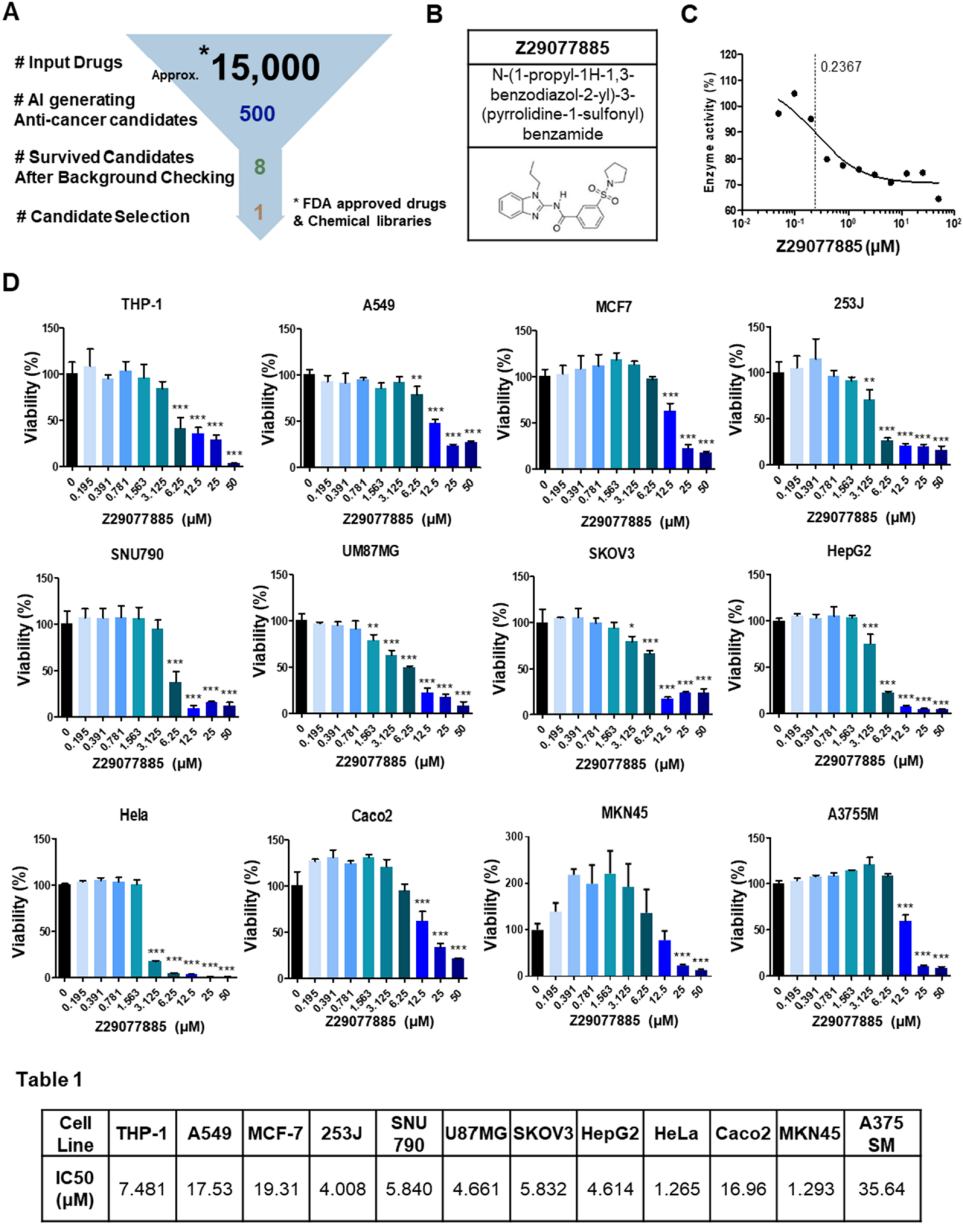
A new anti-cancer drug targeting STK33, Z29077885. **A** Scheme for virtual screening of anti-cancer drugs. **B** Structure of Z29077885. **C** STK33 enzymatic inhibitory function of Z356388010 in vitro. **D** Anti-cancer efficacy on cell viability after Z356388010 treatment in various cancer cell lines in vitro was measured by Cell-titer Glo. **Table 1,** Summary of IC50 of Z29077885 in various cancer cell lines in vitro. Error bars represent SD over biological replicates. The p values were obtained using One-way ANOVA (*p < 0.05, **p < 0.01, ***p < 0.001)

Potential anticancer drugs were selected using an AI-aided drug repurposing module. The module was constructed by training drug response data using the Gradient Boosting Machine (GBM) model to predict potential indications of drugs by providing drug-perturbed gene expression data as input. Consequently, 166,164 drug response data for 1,815 approved drugs were obtained from the LINCS Consortium [15], and the 1,815 drugs were further categorized into 15 indications based on MeSH Therapeutic Uses [16]. The collected drug response data were labeled with the drug indication and used for training. Furthermore, 984,929 drug response data with no labeled indication were separately collected and used as the input of the trained model. After analyzing the results, we chose 500 drugs that scored highly for ‘Antineoplastic Agents.’ Of these, 493 drugs were further investigated to filter out those known to have anticancer effects. Finally, we prioritized eight drugs and validated them experimentally (Fig. 1A and Additional file 1: Fig. S1).

Among the eight anticancer drug candidates, *N*-(1-propyl-1H-1,3-benzodiazol-2-yl)-3-(pyrrolidine-1-sulfonyl) benzamide (Z29077885) was a compound originally developed for a nucleocapsid assembly inhibitor and has never found as an anticancer reagent [17] (Fig. 1B). We found Z29077885 as an anticancer drug targeting STK33 based on the public open bioactivity data related to the compound (Additional file 4: Table S1) [32]. To validate Z29077885 as an anticancer drug targeting STK33, we first tested whether it inhibits the enzymatic function of STK33. The kinase activity of STK33 was measured using treatment with varying concentrations of Z29077885. As shown in Fig. 1C, Z29077885 inhibited STK33 kinase activity, and its AC_50_ was 0.237 μM. However, the inhibitory activity of Z29077885 was about tenfold weaker than that of the known STK33 kinase inhibitors, BRD-8899 (STK33 AC_50_ = 0.011 μM) and ML-281 (STK33 AC_50_ = 0.014 μM) [14]. Once the STK33 kinase inhibitory function of the compound was confirmed, we next tested its anticancer efficacy in various cancer cell lines with various STK33 expressions for a comprehensive understanding of the drug. The viability of various cancer cells decreased with increasing concentrations of Z29077885 (Fig. 1D). Moreover, Z29077885 showed varying IC_50_ values in different cancer types, indicating the specific underlying mechanism associated with its anticancer efficacy (Fig. 1). In comparison to Z29077885 treatment in normal breast cancer cell line MCF-10A (Additional file 2: Fig. S2), Z29077885 treatment in cancer cells decreased more the cell viability (Fig. 1D). Therefore, we determined Z29077885, *N*-(1-propyl-1H-1,3-benzodiazol-2-yl)-3-(pyrrolidine-1-sulfonyl) benzamide, as a new potent anticancer drug targeting STK33.

### Distinct anticancer efficacy of Z29077885

**Fig. 2:**
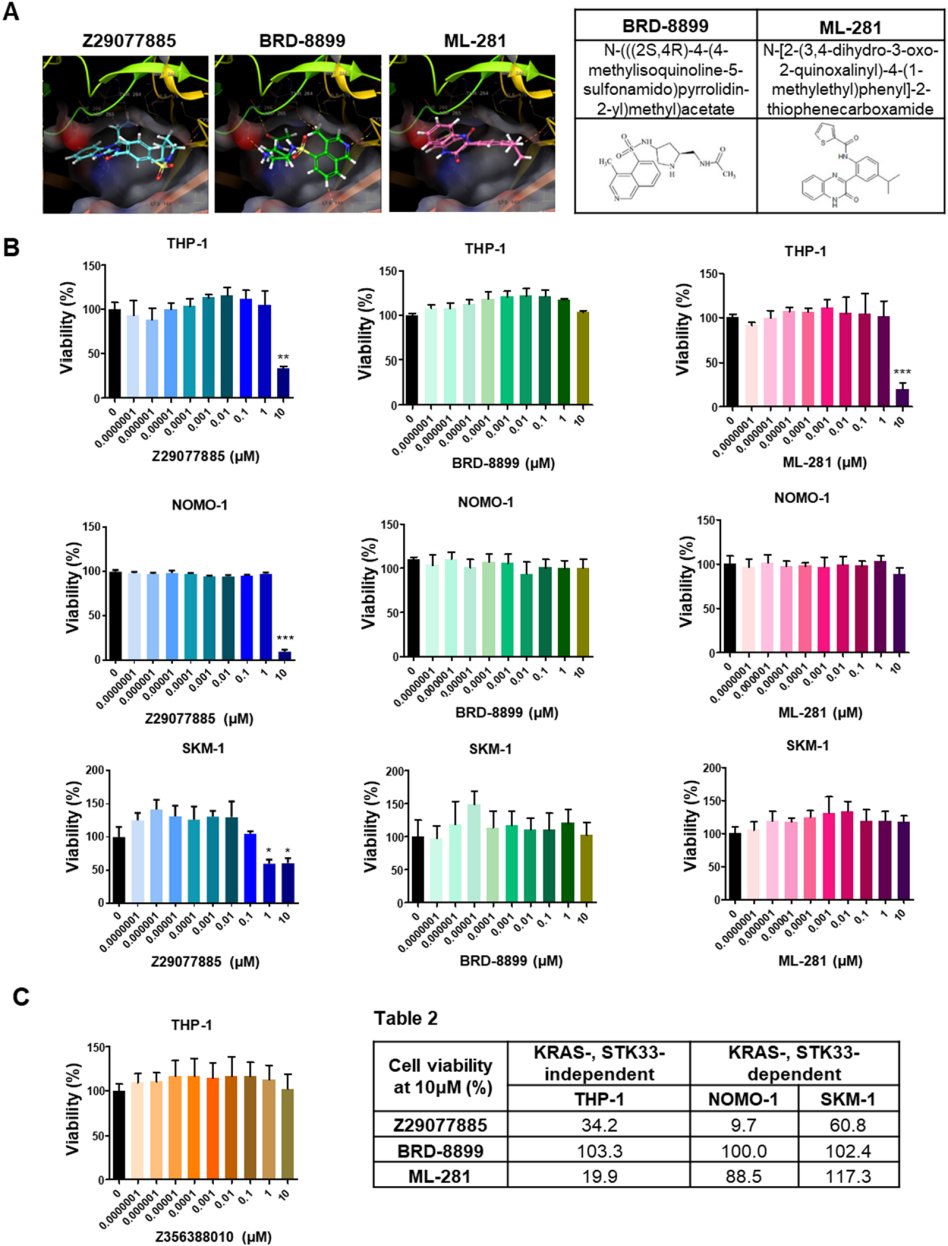
Distinct anti-cancer efficacy of a new STK33 inhibitor, Z29077885. **A** Simulation of docking site at STK33 for Z29077885, BRD-8899, and ML-281. **B** Anti-cancer efficacy on cell viability of Z29077885, BRD-8899, or ML-281 in THP-1, NOMO-1, and SKM-1 cell lines in vitro. **C** Cell viability after Z356388010 treatment with varying concentrations in THP-1 cells in vitro was measured by Cell-titer Glo. **Table 2** Summary of cell viability after 10uM of Z29077885 treatment in vitro. Error bars represent SD over biological replicates. The p values were obtained using One-way ANOVA (*p < 0.05, **p < 0.01, ***p < 0.001)

STK33 as a potential therapeutic target in cancer is controversial since several known STK33 inhibitors neither inhibit cancer cell proliferation nor induce cancer cell death [13, 18]. To further confirm the anticancer activity of Z29077885 and the KRAS and STK33 dependency, comparative experiments with other known STK33 inhibitors, BRD-8899 and ML-281, were performed. First, we predicted the binding site of Z29077885 in STK33 and compared it with the sites in BRD-8899 and ML-281. BRD-8899 and ML-281 interact with the protein kinase domain of STK33; Z29077885 showed a similar binding site at STK33, albeit with a lower docking score, explaining the lower kinase inhibitory activity of Z29077885 (Fig. 2A). Next, THP-1 (KRAS-independent, STK33-independent), NOMO-1 (KRAS-dependent, STK33-dependent), and SKM-1 (KRAS-dependent, STK33-dependent) leukemia cell lines were used to compare the anticancer effect of Z29077885 to those of BRD-8899 and ML-281[13]. In contrast to that with BRD-8899 and ML-281, Z29077885 treatment, at 10 μM, significantly decreased the viability of THP-1 and NOMO-1 cells. Additionally, Z29077885 treatment decreased the viability of SKM-1 cells at 1 μM or more (Fig. 2B). Consistent with a previous study, BRD-8899 treatment did not induce significant changes in cell viability at any concentration; however, ML-281 treatment decreased THP-1 cell viability at 10 μM (Fig. 2B and Table 2). To confirm the specificity of Z29077885, Z356388010 (*N*-(3,4-difluorophenyl)-4-fluoro-3-(pyrrolidine-1-sulfonyl)), another benzamide compound with antiviral activity was tested to see whether it has anticancer efficacy in the cancer cells (International Publication No. WO2014/106019). In contrast to the data shown in Fig. 2B, there were no significant changes in cell viability at any concentration of Z356388010 (Fig. 2C). Altogether, the results indicate that Z29077885 is a potent anticancer drug regardless of *KRAS* mutation or STK33 expression status.

### Z29077885 induces apoptosis and s-phase arrest in cancer cells

**Fig. 3:**
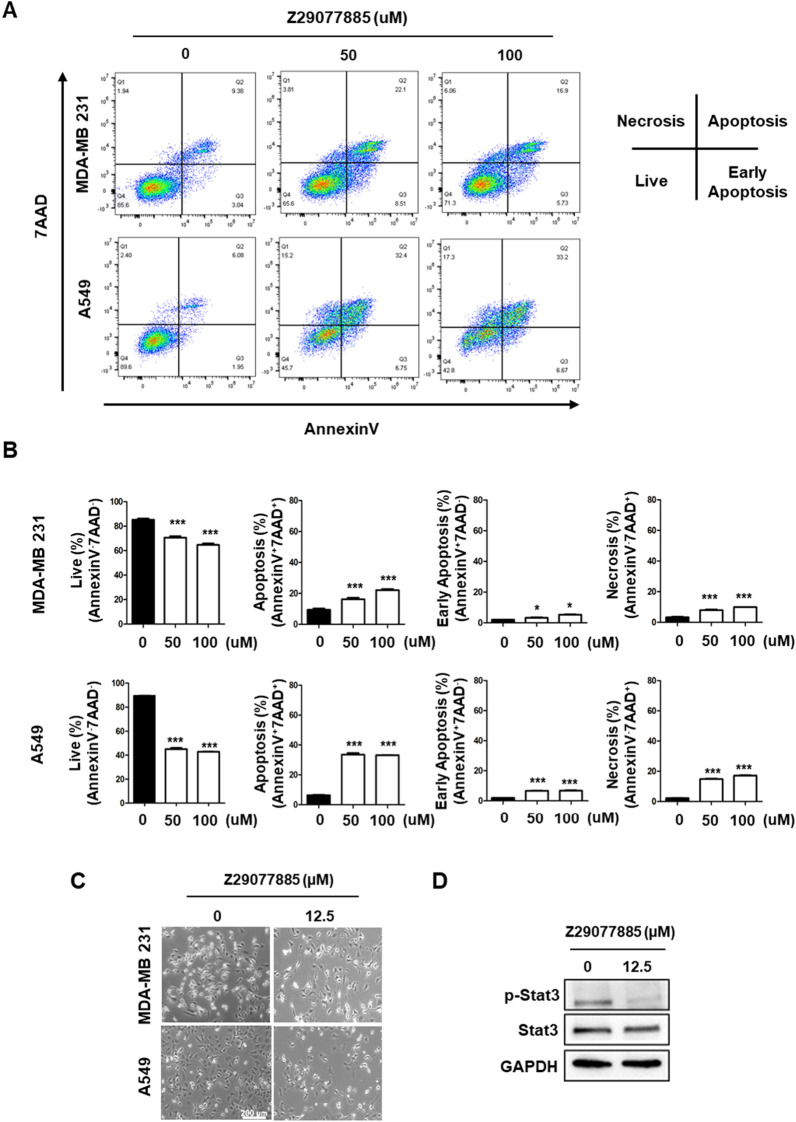
Z29077885 induces apoptosis. **A** and **B** Apoptosis assay for anti-cancer efficacy of Z29077885 in MDA-MB-231 and A549 cell lines was measured by Annexin IV and 7AAD flow cytometry. **C** cell viability of Z29077885 in MDA-MB-231 and A549 cell lines was taken by bright-field microscopy, bars indicated 200. **D** Western blot to test STAT3 signaling deactivation by Z29077885 treatment at 12,5 uM in A549 cells. Error bars represent SD over biological replicates. The p values were obtained using One-way ANOVA (*p < 0.05, **p < 0.01, ***p < 0.001)

**Fig. 4:**
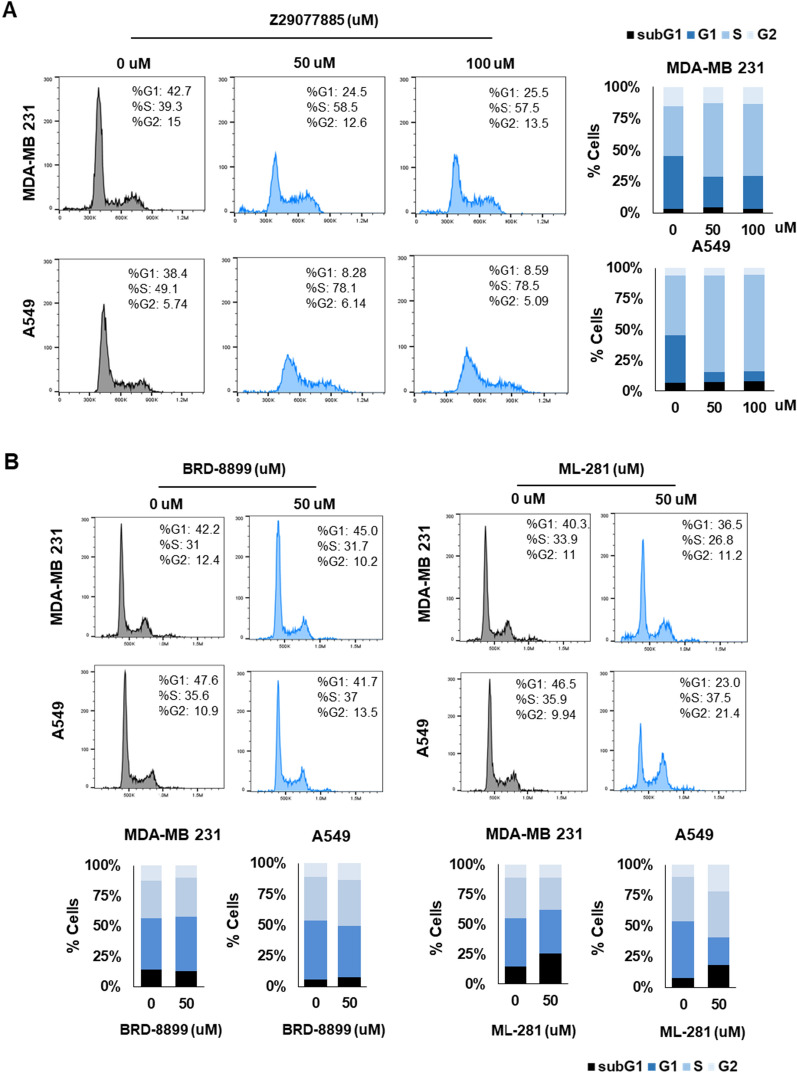
Mechanism of action associated with a new STK33 inhibitor, Z29077885. **A** Cell cycle assay for anti-cancer efficacy of Z29077885 in MDA-MB-231 and A549 cell lines at 50 uM and 100 uM was measured by PI flow cytometry. **B** Cell cycle assay for the effect of BRD-8899 and ML-281 in MDA-MB-231 and A549 cell lines at 50 uM was measured by PI flow cytometry

As Z29077885 showed lower STK inhibitory activity and different characteristics in terms of anticancer efficacy compared to BRD-8899 and MLB-281, we hypothesized that Z29077885 has a distinct molecular mechanism of action related to anticancer efficacy. Therefore, we predicted the mechanism of action of Z29077885 using the public open prediction tool, L1000FWD, which was found to be an inhibition of cyclin-dependent kinase (CDK), with the highest probability of 0.6274 (Additional file 5: Table S2). Next, to analyze the CDK inhibitory function of Z29077885, we first selected model cell lines, MDA-MB-231 breast cancer cells and A549 lung cancer cells, in which the function of STK33 is well understood [19–21]. To confirm the predicted anticancer mechanism of Z29077885 in MDA-MB-231 and A549 cells, we measured their apoptosis upon drug treatment using flow cytometer analysis. The cell viability was decreased and apoptosis was enhanced upon treatment of both the cell lines with Z29077885 (Fig. 3A and B). Inhibition of CDK activity blocks STAT activation [22, 23]. Therefore, we next confirmed the reduced cell viability and STAT3 deactivation in A549 cells upon 12.5 μM of Z29077885 treatment (Fig. 3C and D). As CDKs play important roles in cell division control [24, 25], we next analyzed the cell cycle following treatment of MDA-MB-231 and A549 cells with Z29077885 and found that the cell cycle was arrested at the S phase, supporting both the predicted mechanism of action after Z29077885 treatment (Fig. 4A). Inconsistent with the effect of Z29077885 on cell cycle arrest at the S phase, neither BRD-8899 nor ML-281 induced cell cycle arrest at the S phase in MDA-MB-231 and A549 cells (Fig. 4B). Interestingly, we found ML-281-induced cell cycle arrest at the G2 phase, explaining the reduced cell viability upon 10 μM of the drug treatment in THP-1 cells (Fig. 2B and Fig. 4B). In summary, we found that Z29077885 induces apoptosis via deactivation of the STAT3 signaling pathway, and induces cell cycle arrest at the S phase which is a different mechanism of action compared to known STK33 inhibitors.

### Potential of Z29077885 as a cancer therapeutics in vivo

**Fig. 5:**
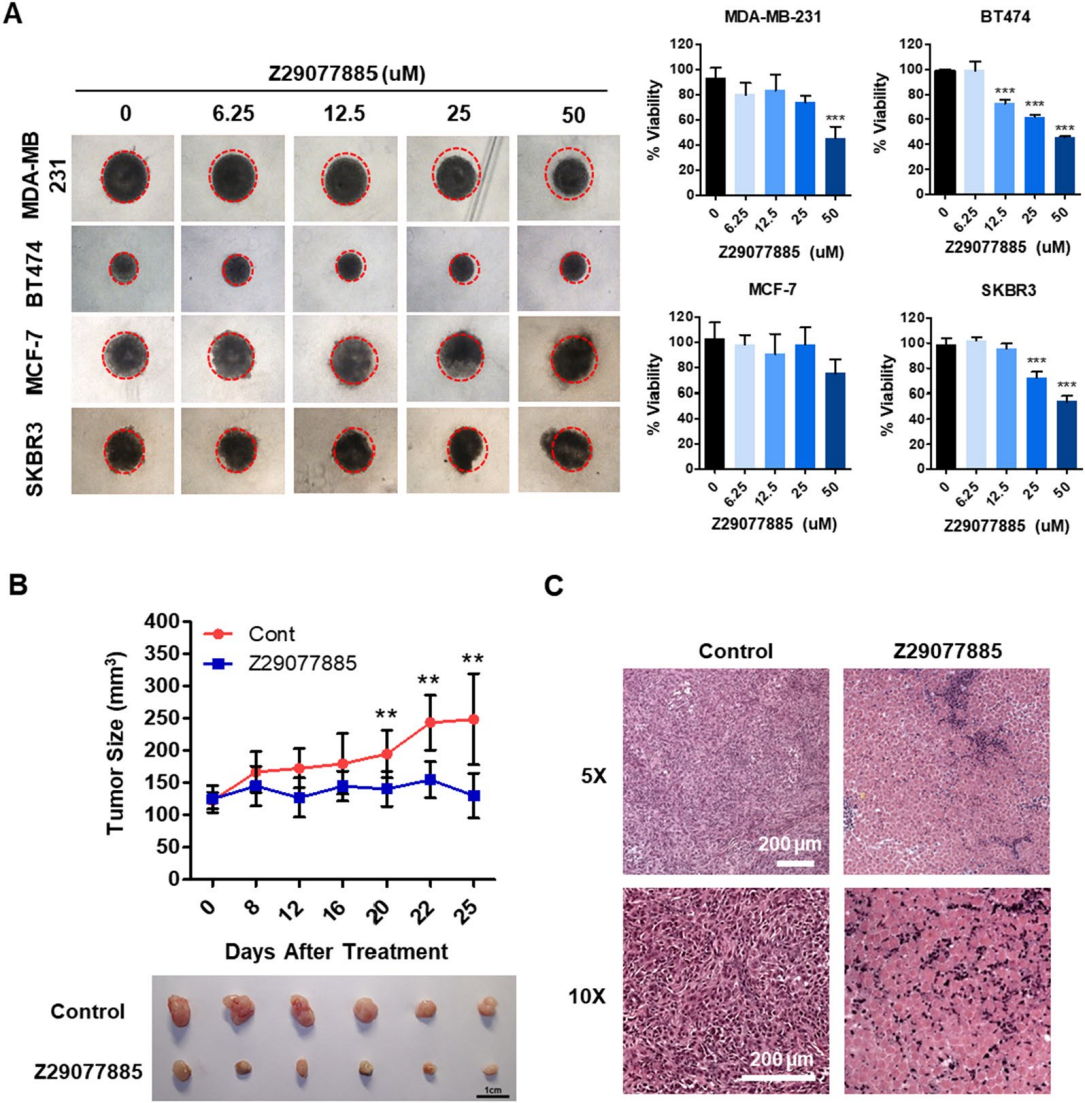
The potential of Z29077885 as therapeutics for cancer treatment in vivo. **A** Anti-cancer efficacy of Z29077885 3D in vitro MDA-MB-231, BT474, MCF-7, and SKBR3 cell lines was taken by bright-field microscopy(right), cell viability was tested by Cell-titer Glo (left). **B** Z29077885 treatment showed anti-tumor efficacy by reducing tumor size in vivo A549 xenograft. **C** Enhanced necrosis by Z29077885 treatment in vivo shown by immunohistochemistry. Bars indicated 200 μm. Error bars represent SD over biological replicates. The p values were obtained using One-way ANOVA (*p < 0.05, **p < 0.01, ***p < 0.001)

To test the potential of the new STK33 inhibitor Z29077885 as a therapeutic, we first selected breast cancer cell lines in which the function of STK33 is well understood, next generated three-dimensional (3D) breast cancer spheroids known to mimic cancer physiology [26, 27] and treated them with the drug for 72 h. MDA-MB-231, BT474, and SKBR3 spheroids showed significant decreases in cell viability at 50 μM concentration, whereas MCF-7 cells did not (Fig. 5A). Among these spheroids, Z29077885 showed better efficacy in the MDA-MB-231 and BT474 spheroids than in the SKBR3 spheroids. The size of the MDA-MB-231 and BT474 spheroids reduced with increasing concentrations of Z29077885, whereas the SKBR3 spheroids showed broken clustering, indicating an increase in their apoptotic cell population (Fig. 5A left and Additional file 3: Fig. S3). Even though it seems that the cancer cells showed drug-affected morphologies, we are not able to evaluate dying cells by microscope images showing the size of the spheroid. Therefore, we performed a cell viability assay to accurately evaluate drug efficacy (Fig. 5A). Next, we tested the anti-tumor efficacy of Z29077885 in vivo. After inoculation, A549 cells were first treated with Z29077885 for 8 days, followed by two additional injections on days 4 and 8 after the first treatment. Z29077885 treatment decreased the tumor size in A549 lung cancer xenograft in vivo (Fig. 5B). In addition, we observed necrotic areas using hematoxylin and eosin (H&E) staining (Fig. 5C). Therefore, we confirmed Z29077885 as a new anticancer agent targeting STK33 in vitro and in vivo, discovered using the AI deep learning method; this might provide clinical benefits during cancer treatment.

## Discussion

In the present study, we found that the benzamide compound Z29077885 functions as an anticancer drug, using AI deep learning. We demonstrate that Z29077885 can inhibit STK33 enzymatic function in vitro and decrease cell viability regardless of the *KRAS* mutation status and STK33 expression level in cancer cells. In addition, Z29077885 shows anti-tumor efficacy in vivo. Finally, we confirmed cell cycle arrest at the S phase as the underlying mechanism of action of Z29077885. In this study, the results proved that drug repurposing using the AI deep learning method in our study was successful. However, the STK33 inhibitory function of Z29077885 associated with anticancer efficacy is necessary in the next study.

Although the STK33 inhibitor was the main objective of our research, the prediction results (Additional file 4: Table S1) indicated that the compound also exhibited AURKB inhibition. We plan to conduct additional experiments to investigate the AURKB effect. As our research is still in the early stages, no negative side effects have yet been identified. Moving forward, we aim to conduct additional experiments to examine not only the additional effects of drugs but also their side effects.

Drug repurposing is mostly accidental and has numerous outcomes. The successes have encouraged the development of systemic approaches, such as computational approaches [28]. AI-driven drug repurposing is beneficial for developing new therapeutic strategies against diseases. In particular, repurposing existing non-cancer drugs for cancer therapy is an attractive strategy because of the low risk of safety failure and less time and cost consumption [29–31]. Moreover, AI-driven drug repurposing can be an effective tool for rapid and tangible approaches during an emergency such as the current COVID-19 crisis [32, 33]. Owing to its capability of simultaneous processing of a large amount of data, the virtual screening strategy is useful for the rapid discovery of drug candidates for any known target protein. Furthermore, AI can also play an important role in designing appropriate combinations of drugs with different targets and pathways [34]. Even though the new anticancer drug discovered in this study needs to be further evaluated for it to progress to the next stage of development, we believe it has potential as a new and promising therapeutic strategy against cancer in combination with different drugs.

## Conclusion

In this study, we identified a new anticancer drug targeting STK33 that induces apoptosis and cell cycle arrest at the s phase using an AI-driven screening method. We also found that Z29077885 treatment inhibits cancer cell growth and induces necrosis in an in vivo A549 xenograft. Even though the STK33 inhibitory function of Z29077885 associated with the anticancer effect needs to be further investigated, a new anticancer drug repurposed by AI can be a promising therapeutic agent against cancers.

### Supplementary Information

**Additional file 1: Fig. S1.** Test of anti-cancer efficacy of 7 prioritized drugs as anti-cancer agents by the virtual screening. Anti-cancer efficacy on cell viability after drug treatment in various cancer cell lines in vitro was measured by Cell-titer Glo. Error bars represent SD over biological replicates. The p values were obtained using One-way ANOVA (*p < 0.05, **p < 0.01, ***p < 0.001).

**Additional file 2: Fig. S2.** Test of Z29077885 toxicity on normal breast cancer cell line MCF 10A. Error bars represent SD over biological replicates. The p values were obtained using One-way ANOVA (*p < 0.05, **p < 0.01, ***p < 0.001).

**Additional file 3: Fig. S3.** Cancer spheroid morphology under a higher magnificent of microscope.

**Additional file 4: Table S1.** Bioactivity of Z29077885 from the public database, Pubchem.

**Additional file 5: Table S2.** Predicted MOA for Z29077885. L1000FWD visualization of drug-induced signatures. Signatures are colored by the mechanism of action (MOA)

## Data Availability

The datasets used and/or analysed during the current study are available from the corresponding author on reasonable request.
