# Supplementary figures and images for: Artificial intelligence-driven new drug discovery targeting serine/threonine kinase 33 for cancer treatment
Source: Cancer Cell Int. 2023 Dec 12;23:321. doi: 10.1186/s12935-023-03176-2 (PMC10717841; doi:10.1186/s12935-023-03176-2)

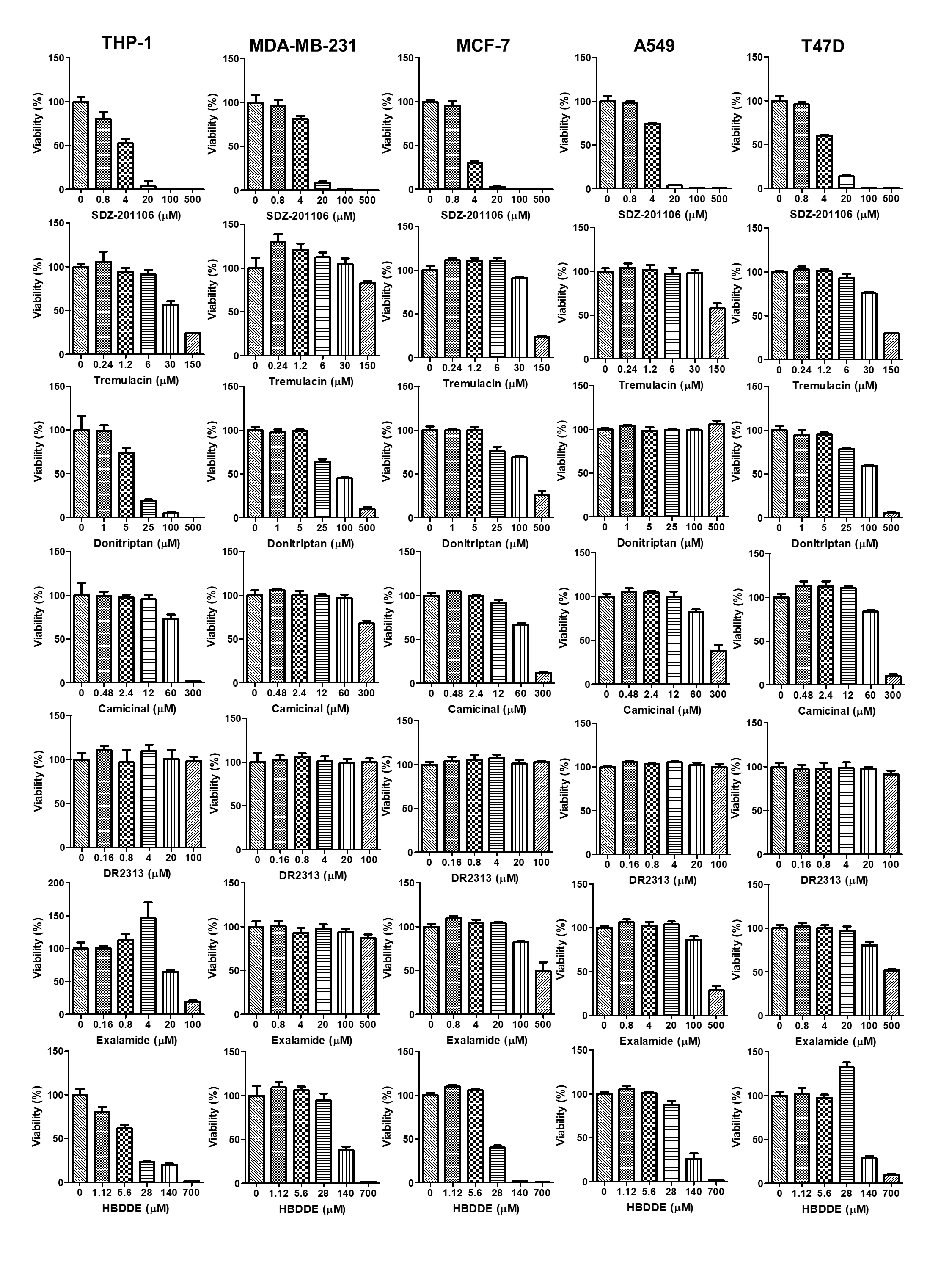

Supplement: Supplementary file 1 — Additional file 1: Fig. S1. Test of anti-cancer efficacy of 7 prioritized drugs as anti-cancer agents by the virtual screening. Anti-cancer efficacy on cell viability after drug treatment in various cancer cell lines in vitro was measured by Cell-titer Glo. Error bars represent SD over biological replicates. The p values were obtained using One-way ANOVA (*p < 0.05, **p < 0.01, ***p < 0.001). [file 12935_2023_3176_MOESM1_ESM.tif]

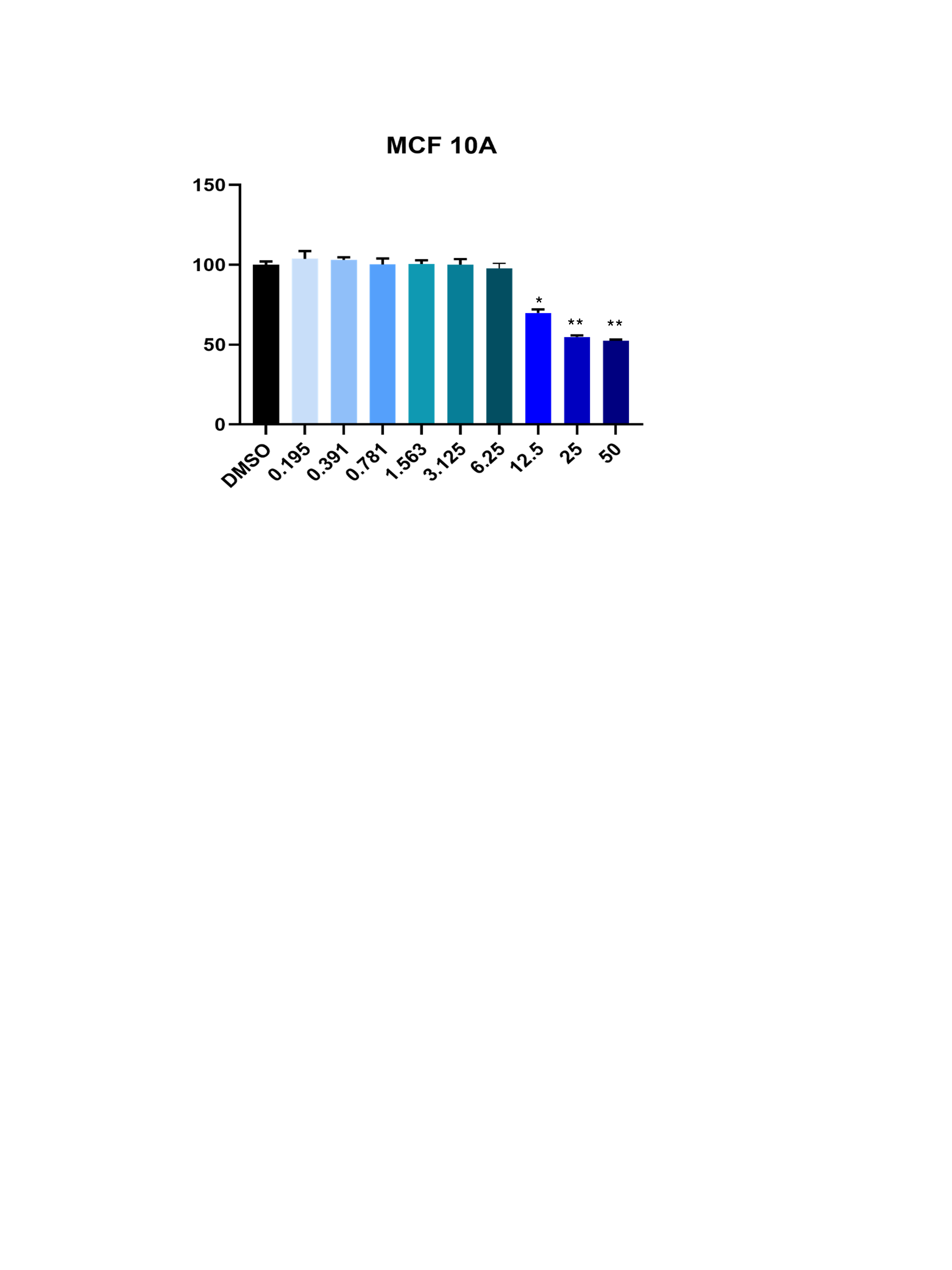

Supplement: Supplementary file 2 — Additional file 2: Fig. S2. Test of Z29077885 toxicity on normal breast cancer cell line MCF 10A. Error bars represent SD over biological replicates. The p values were obtained using One-way ANOVA (*p < 0.05, **p < 0.01, ***p < 0.001). [file 12935_2023_3176_MOESM2_ESM.tif]

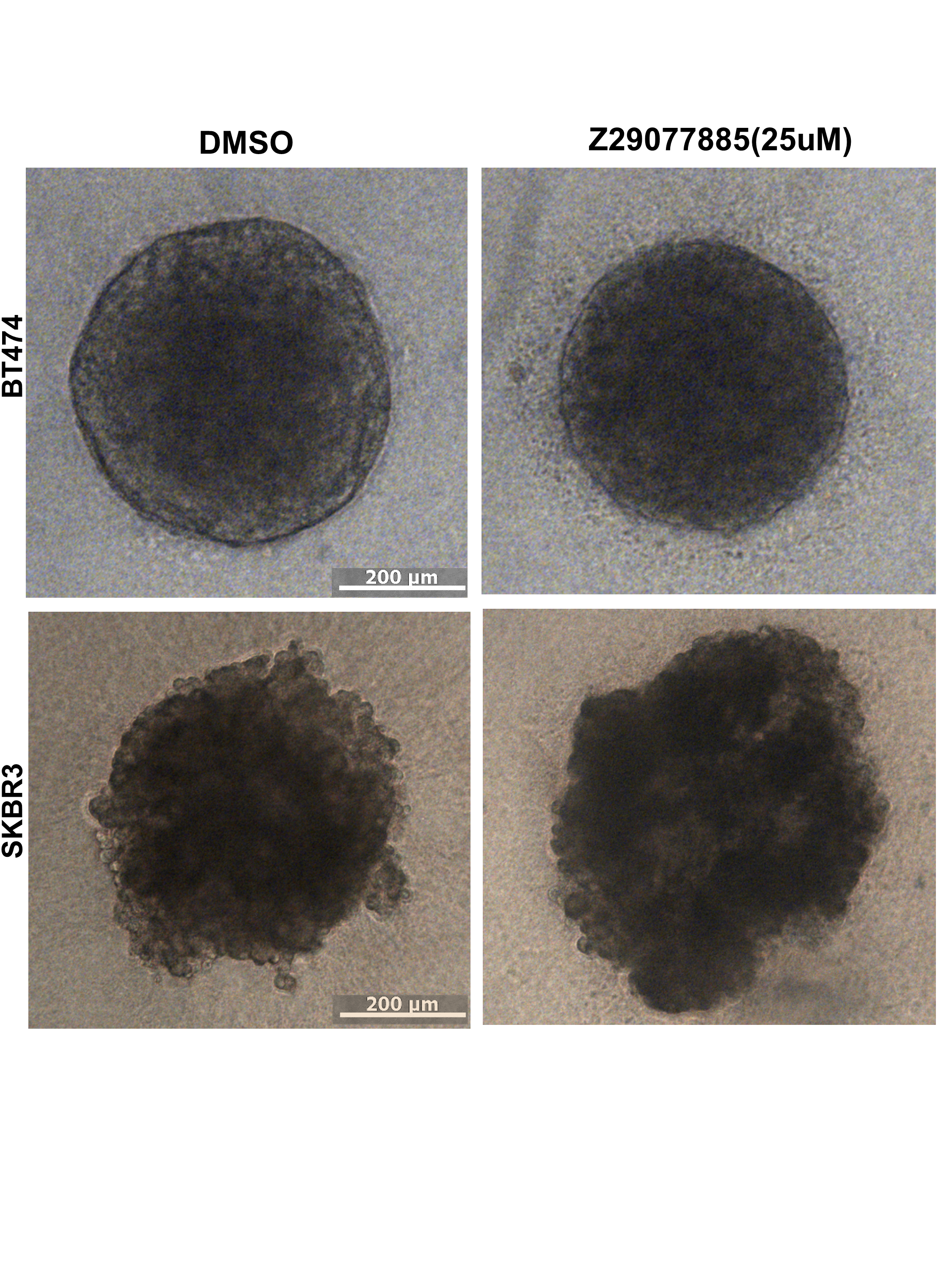

Supplement: Supplementary file 3 — Additional file 3: Fig. S3. Cancer spheroid morphology under a higher magnificent of microscope. [file 12935_2023_3176_MOESM3_ESM.tif]

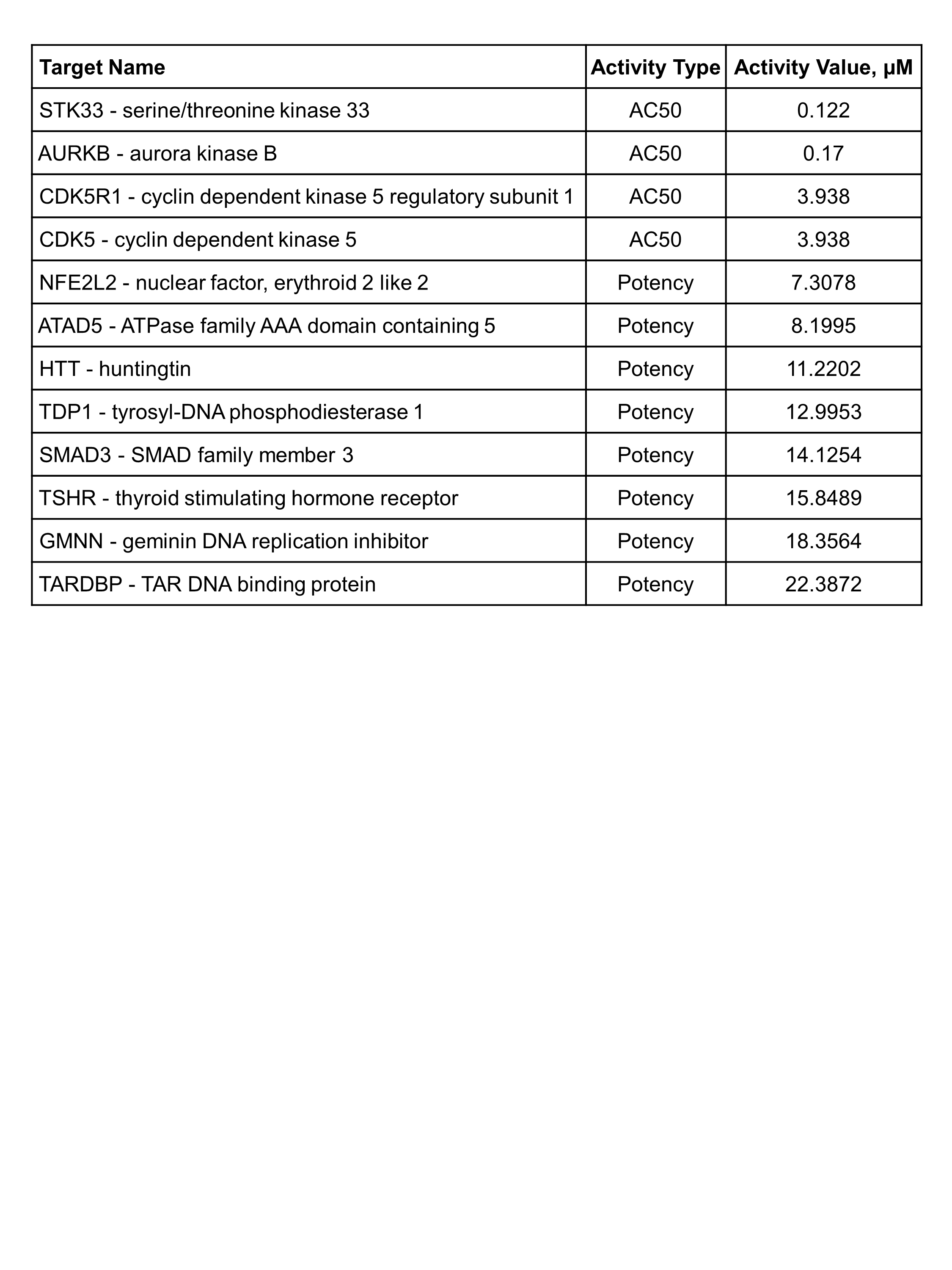

Supplement: Supplementary file 4 — Additional file 4: Table S1. Bioactivity of Z29077885 from the public database, Pubchem. [file 12935_2023_3176_MOESM4_ESM.tif]

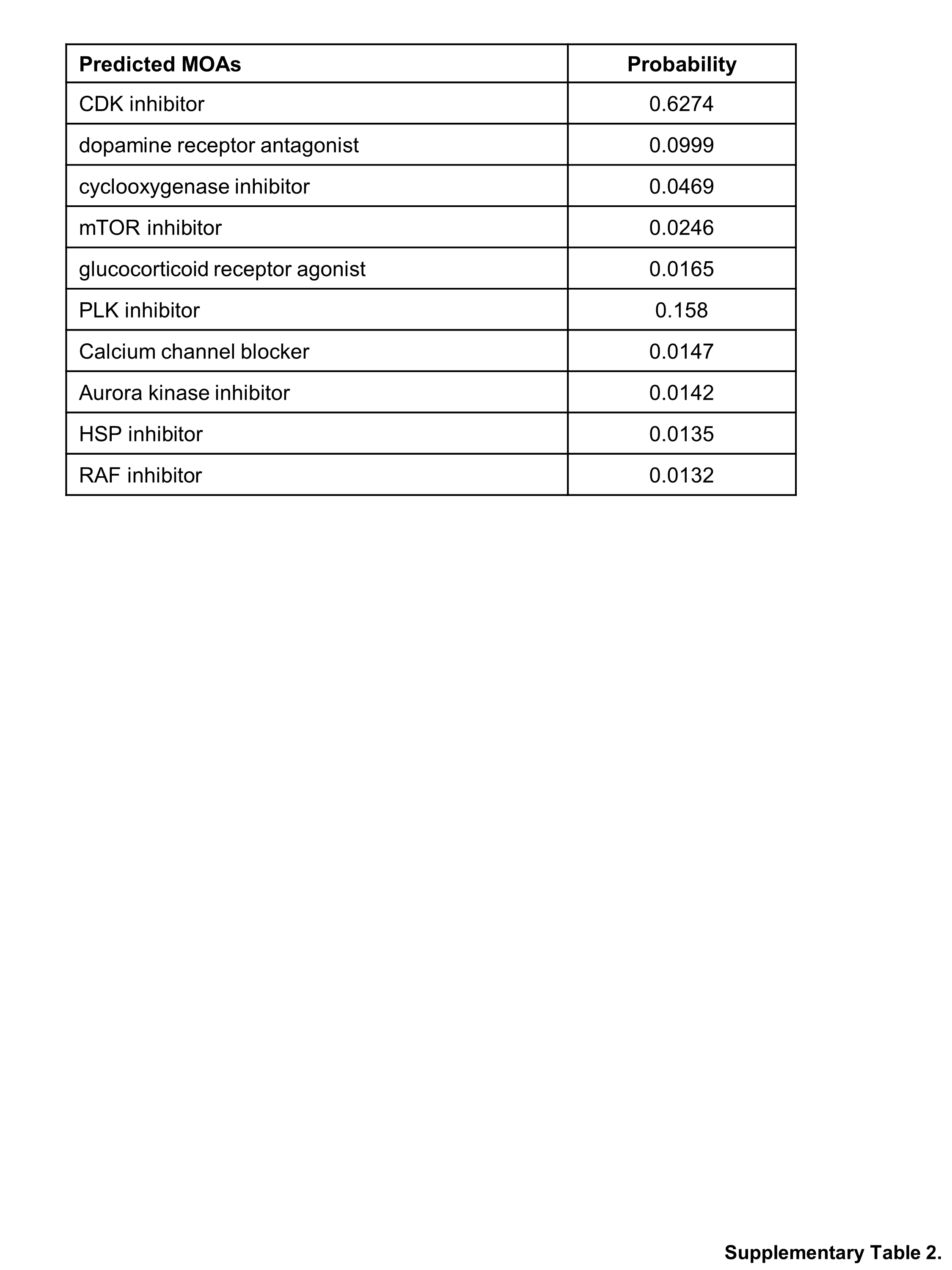

Supplement: Supplementary file 5 — Additional file 5: Table S2. Predicted MOA for Z29077885. L1000FWD visualization of drug-induced signatures. Signatures are colored by the mechanism of action (MOA) [file 12935_2023_3176_MOESM5_ESM.tif]
